# Reduced neural sensitivity to social stimuli in infants at risk for autism

**DOI:** 10.1098/rspb.2012.3026

**Published:** 2013-05-07

**Authors:** S. Lloyd-Fox, A. Blasi, C. E. Elwell, T. Charman, D. Murphy, M. H. Johnson

**Affiliations:** 1 Centre for Brain and Cognitive Development, Birkbeck, University of London, Malet Street, London WC1E 7HX, UK; 2 Department of Medical Physics and Bioengineering, University College London, London, UK; 3 Department of Psychology, Institute of Psychiatry, Kings College London, London, UK; 4 Department of Forensic and Neurodevelopmental Science, Institute of Psychiatry, Kings College London, London, UK

**Keywords:** functional near infrared spectroscopy, social, infancy, development, autism spectrum disorder, voice processing

## Abstract

In the hope of discovering early markers of autism, attention has recently turned to the study of infants at risk owing to being the younger siblings of children with autism. Because the condition is highly heritable, later-born siblings of diagnosed children are at substantially higher risk for developing autism or the broader autism phenotype than the general population. Currently, there are no strong predictors of autism in early infancy and diagnosis is not reliable until around 3 years of age. Because indicators of brain functioning may be sensitive predictors, and atypical social interactions are characteristic of the syndrome, we examined whether temporal lobe specialization for processing visual and auditory social stimuli during infancy differs in infants at risk. In a functional near-infrared spectroscopy study, infants aged 4–6 months at risk for autism showed less selective neural responses to social stimuli (auditory and visual) than low-risk controls. These group differences could not be attributed to overall levels of attention, developmental stage or chronological age. Our results provide the first demonstration of specific differences in localizable brain function within the first 6 months of life in a group of infants at risk for autism. Further, these differences closely resemble known patterns of neural atypicality in children and adults with autism. Future work will determine whether these differences in infant neural responses to social stimuli predict either later autism or the broader autism phenotype frequently seen in unaffected family members.

## 1. Introduction

In the hope of discovering early markers of autism, attention has recently turned to the study of infant siblings of children with autism [1–3]. Because the condition is highly heritable, later-born siblings of diagnosed children are at substantially higher risk for developing autism or the broader autism phenotype than the general population [4,5]. While the majority of these infants at risk do not go on to autism, studies have shown that first-degree family members can evidence traits—in visual and auditory perception—that overlap with those observed in autism [6]. Several studies have attempted to differentiate the at-risk infants who subsequently receive a clinical diagnosis from those who do not, as well as comparing them with low-risk infants with no family history of autism. Moreover, prospective longitudinal studies of infants at risk allows one to search for ‘endophenotypes’ for autism—intermediate phenotypes (often aspects of brain structure and function) between genes and the resulting clinical phenotype [7]—given that during development they should be present prior to the appearance of full clinical symptoms [8]. Current evidence indicates that infants who go on to receive a diagnosis as toddlers begin to be identified from around the end of the first year on the basis of atypical social and non-social behaviours such as unusual eye contact, lack of orientation to name and reduced flexibility in switching attention [1,2,9]. There is growing consensus that these early behavioural manifestations of autism are subtle and that these clinically detectable symptoms emerge gradually during development [1]. Thus, there is an active search for early neural markers that may precede or predict the later emergence of behavioural symptoms. A cautionary note is that while group-level differences have been reported, to date no studies have reported sensitivity and specificity of individual markers to later autism outcomes that could have clinical utility.

Converging evidence has implicated atypical social perception (i.e. processing of cues which allow us to interpret the intentions and dispositions of others [10]) and brain function in children and adults diagnosed with autism [11]. However, the developmental causes of this atypical neural phenotype remain unknown. In typical adults, a network of regions termed the ‘social brain’ [12] have been identified, which include the orbitofrontal cortex, amygdala, temporal lobe face-sensitive regions and superior temporal sulcus (STS) region (hereafter we use the term ‘STS region’ to refer to regions of the superior temporal gyrus, sulcus and middle temporal gyrus extending to the temporo-parietal junction). It has been suggested that a problem in one or more of the underlying mechanisms that bias infants to orient towards and attend to socially relevant information from early in life may disrupt the typical developmental trajectory of the social brain network [13–15]. For example, atypical neural responses to face and/or eye contact [16] may interfere with the emergence of critical developmental milestones relevant for later social cognitive skills, such as joint attention. These cascading influences may eventually preclude the typical development of socio-communicative skills.

One perspective on the typical development of the social brain network is that it emerges as a result of processes of ‘interactive specialization’, in which the adult pattern of cortical specialization becomes evident in development through a process of increasing functional specialization (or tuning) of the response of cortical areas [17,18]. According to the interactive specialization view, biases in attention and processing in early infancy are reinforced by differential patterns of experience, subsequently resulting in the patterns of cortical specialization associated with the social brain observed in adults. In autism, disruption in the typical emergence of the social brain network may arise due to an atypical early trajectory, which then becomes compounded by atypical interactions with the environment, leading to the well-established pattern of symptoms of autism becoming embedded and observable by the age of diagnosis [14].

Several previous studies have identified differences in brain function in response to socially relevant stimuli between infants with low and high risk of developing autism [16,19–22]. We use the term ‘social’ in this paper in the broadest sense (i.e. that they are human-generated cues, either visual or auditory, which originate from conspecifics). This does not necessarily imply that these cues are intended to be communicative. One of the first such studies of visual social stimuli using electroencephalography (EEG) found that compared with low-risk controls, high-risk infants of 10 months of age showed delayed neural responses to faces with direct gaze [16]. More recently, related effects have been associated with a later diagnosis of autism at 3 years of age [6]. In a study of infants under 12 months, McCleery *et al*. [20] found that pictures of toys elicited significantly faster neural responses in high-risk infants compared with pictures of faces, while in the low-risk age-matched infants the pattern was reversed. However, another recent study on face perception of familiar and unfamiliar faces with high-risk infants of 12 months of age did not find any striking group differences compared with low-risk controls [19].

While recent neuroimaging research has shown that portions of the temporal lobe can be selectively activated by social auditory stimuli (human vocal sounds) and non-social environmental sounds in 4–7-month-old human infants [23–26], functional magnetic resonance imaging (fMRI) research in adults with autism failed to identify vocal selective regions of the STS [27]. In accordance with these findings, evidence in adults suggests that individuals with autism have difficulties in vocal perception, such as impairment in the attribution of mental state within a voice [28] and a lack of preference for their mother's voice [29]. Moreover, EEG findings in children with autism suggest a selective impairment in attention to vocal-speech sounds [30], and recent magnetic resonance imaging (MRI) work has identified a lack of left temporal specialization for language in 1–4-year-olds [31]. Therefore, examining the brain correlates associated with processing social stimuli at an earlier age may help define the infant autism endophenotype further.

To date, the study of brain function in infant siblings of children with autism has relied heavily on EEG. However, it remains challenging to accurately localize the cortical generators of scalp-recorded EEG in infants owing to its susceptibility to data corruption by movement artefacts [32], volume currents and current lack of infant head models [33]. Functional near-infrared spectroscopy (fNIRS) potentially provides an ideal method for improving our current understanding of cortical activity in the early developing atypical brain as it can be widely adopted owing to its relatively low cost, ease of use with infants, capacity for more specific spatial localization with respect to EEG and suitability for use in naturalistic settings [34–36]. Though the depth resolution of fNIRS is dependent on the age of the infant and the optical properties of the tissue [37], and offers lower spatial resolution relative to fMRI, it is similar in that it measures haemodynamic responses to neuronal activation. Research from adults has shown a high degree of correlation between simultaneous recordings of haemodynamic responses with fNIRS and fMRI [38]. Thus, the data acquired from fNIRS can complement the high spatial resolution of function and anatomy data obtained with MRI [31,39].

In previous work, we have used fNIRS to show that specific regions of the frontal and temporal lobes in 4–7-month-old human infants are selectively activated by visual and auditory social stimuli relative to non-human stimuli in 5-month-old infants [25,40,41]. In the present study, we used an fNIRS protocol from previous work [25] to ascertain the extent to which infants at risk for autism show early specialization of the frontal and temporal cortex for the processing of social stimuli (visual social stimuli such as ‘peek-a-boo’, and auditory social stimuli such as yawning, coughing and laughing). While not all infants from the group at risk will go on to a diagnosis of autism, the majority of the group may show trait neural signatures [6] that, when combined with other factors, can result in later-emerging autism. Specifically, we hypothesized that, in comparison with *low-risk* infants, the group of infants at risk for autism (*high-risk*) would show less evidence of temporal lobe areas being tuned to social visual and auditory stimuli. This would be evident as fewer channels showing evidence of vocal-specialized activation (*vocal* > *non-vocal*) in the temporal lobe of the high-risk group when compared with the low-risk group. Further, we would expect fewer channels over the temporal lobe to evidence significant responses to the *visual social* stimuli in relation to the visual non-social baseline in the high-risk group compared with the low-risk group. In addition, we were also interested in investigating non-vocal selective responses given the discrepancy between findings in infants evidencing non-vocal selective responses in the posterior superior temporal region [23,25] compared with adults with [42] and without autism [27], which show a marked absence of this response.

## 2. Material and methods

### (a) Participants

Thirty-four 4–6-month-old infants participated in this study, comprising 18 infant siblings of children with autism (high-risk; 10 female, mean age = 149.56 days, s.d. = 26.75) and 16 infants who have no family history with autism (low-risk; 6 female, mean age = 153.81, s.d. = 25.67; note that 14 of the low-risk infants contributed data to a previous study on voice processing [25]). The high-risk infants were from the British Autism Study of Infant Siblings (BASIS; www.basisnetwork.org), all of whom had an older full sibling with a community clinical diagnosis of autism [43]. Families enrol from various regions of the UK, and they are invited to attend multiple research visits over their children's first years of life. Each visit lasts a day or two and is adapted to meet the families’ needs. Measures collected are anonymized and shared among scientists to maximize collaborative value and to minimize burden on the families. A clinical advisory team of senior consultants works closely together with the research team(s) and, if necessary, with the family's local health services, to ensure that any concerns about the child arising during the study are adequately addressed. The low-risk infants were from a volunteer database with no reported family history (first-degree relative) of autism and had at least one older full sibling. The fNIRS data presented in the current study originates from the first two-day visit that the infants attended as part of the prospective long-term project at the Centre for Brain and Cognitive Development. In addition to the fNIRS session, this first visit also included an MRI session (on the second day) and the Mullen Scales for Early Learning [44]. A further 20 infants participated but were excluded from the study (11 high-risk infants, 9 low-risk infants) owing to an insufficient number of valid trials according to looking time measures (14 infants), equipment failure (5 infants) or a high level of rejected data (3 infants; artefact detection algorithms and analyses). This attrition rate is within the typical range for infant fNIRS studies [34] as an exclusion rate (owing to unsufficient looking time) of 10–15 per cent per condition is observed in the majority of fNIRS studies with awake infants (therefore, in the current study the use of three conditions with the same visual stimuli may have contributed to the reported attritrion rate). The infants in the high-risk group fell within the average range of functioning as verified by the Mullen Early Learning Composite score (mean = 102.5; s.d. = 12.69), as did the infants in the low-risk group (mean = 99.44; s.d. = 8.51).

### (b) Experimental procedures

**Figure 1.**
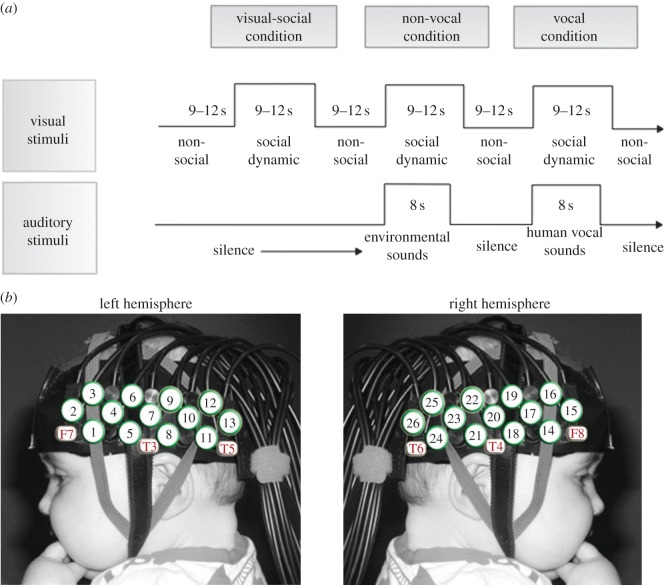
Illustrations of the procedure used in this experiment. (*a*) The experimental design showing the order and timing of stimulus presentation for the three conditions (visual social, vocal and non-vocal). The baseline period is extracted from the sections with no sound and non-social visual stimuli. (*b*) A participant wearing the fNIRS headgear with channel locations and the locations of the 10–20 coordinates on an average 4–6-month-old head displayed. (Online version in colour.)

Infants wore custom-built fNIRS headgear consisting of two source–detector arrays (figure 1), containing a total of 26 channels (source–detector separations: 2 cm), and were tested with the UCL topography system [45]. This system used two continuous wavelengths of source light at 770 and 850 nm. The different channel separations allowed the measurement of activation at different depths into the cortex. Based on an understanding of light transport and given that the cortex is approximately 0.5 cm from the skin surface in this age group (measure taken from structural MRIs) [46], the channel separation used in the current study was predicted to penetrate up to a depth of approximately 1 cm from the skin surface, potentially allowing measurement of both the gyri and parts of the sulci near the surface of the cortex. Before the infants began the study, head measurements were taken to align the headgear with 10–20 coordinates [25]. Measurements from this group of infants showed that the average head circumference was 42.99 cm, and the average distance from the glabella to the ear (T3/T4 of the 10 : 20 system; figure 1) was 11.27 cm (s.d. = 0.72). Therefore, across the majority of the infants, the position of the channels varied relative to T3/T4 by no more than 1 cm. Furthermore, the head measurements did not differ across the two groups (table 1). With the use of age-appropriate infant structural MRIs, anatomical scalp landmarks and the 10–20 system, we can therefore approximate the location of underlying cortical regions for the infants and draw comparisons of general regional activation in infants at risk for autism with findings from adult populations.

**Table 1.** Characteristics of participants included in the analysis and their behaviour during the task. Note that when an entry includes a bracketed number this refers to the s.d. while the first number refers to the mean value across the group.

	low-risk	high-risk
*n*	16	18
age (days)	153.81 (25.67)	149.56 (26.75)
female : male	6 : 10	10 : 8
total number of trials presented	13.77 (2.31)	14.13 (2.96)
total valid trials	12.54 (2.63)	12.06 (2.46)
looking time per trial (%)	94.18 (3.2)	91.07 (5.52)
valid trials in social visual condition	4.38 (0.87)	4.06 (1.03)
valid trials in non-vocal condition	4.08 (1.04)	4.29 (0.92)
valid trials in vocal condition	4.08 (0.95)	4.06 (1.09)
excluded channels/condition	1.67 (1.12)	3.06^a^ (2.81)
Mullen standard score	99.44 (8.51)	102.5 (12.69)
head circumference (cm)	43.3 (1.81)	42.83 (1.59)

^a^Though there was a trend for a significant difference in the number of excluded channels between the high-risk and low-risk groups (*p* = 0.074), this is explained by a particularly high number of excluded channels in two of the infants in the high-risk group (note that if these infants are excluded from the dataset the pattern of significant effects described in this paper do not change).

Once the fNIRS headgear was placed on their heads, the infants sat on their parent's lap in a dimly lit and sound-attenuated room. The parent was instructed to refrain from interacting with the infant during the stimuli presentation unless the infant became fussy or sought their attention. The sequence of stimulus presentation is illustrated in figure 1. The conditions alternated one after the other, with a period of baseline between each. The three types of condition—*visual social* (silent; V-S), *auditory vocal* (V) and *auditory non-vocal* (N-V)—were presented in the same order across infants in a repeating loop (V-S, N-V, V, V-S, V, N-V) of trials (single presentation of a condition) until the infants became bored or fussy, as judged by the experimenter who was monitoring their behaviour. The reference for the haemodynamic change observed in response to the different conditions was obtained from the baseline (described in the following section). Therefore, the resulting activation was specific to the nature of the stimuli (or the contrast of two auditory stimuli) rather than visual or auditory stimulation *per se*.

A restriction of studying auditory processing in awake infants of this age is that they need to be presented with concurrent visual stimulation to reduce infant movement, and thus artefact in the signal. We chose to use the same visual stimuli during the presentation of the auditory stimuli, bearing in mind that we collected data from the same stimulus without auditory stimulation.

#### (i) Visual stimuli

Visual stimuli consisted of full-colour, life-size (head and shoulders only) social videos of female actors who either moved their eyes left or right or performed hand games —‘peek-a-boo’ and ‘incy wincy spider.’ Two visual social videos were presented for varying duration over each 9–12 s trial to avoid inducing anticipatory brain activity. To control for effects of attention—given that the social visual stimuli was sometimes presented simultaneously with auditory stimuli—there were six different visual social videos (two actors; three types of social video), whereas each auditory condition used two different recordings (two speakers; one recording each—see below). During the baseline, visual stimuli were displayed, which consisted of full-colour still images of different types of transport (i.e. cars and helicopters) presented randomly for a pseudorandom duration (1–3 s) for 9–12 s (see figure 1 for stimulus presentation order). Dynamic non-social baseline stimuli have also been used in previous work investigating responses to visual social dynamic stimuli, and have been found to produce similar effects to the static non-social baseline used in the current study [40,41]. These visual stimuli were displayed on a 117 cm plasma screen with a viewing distance of approximately 100 cm.

#### (ii) Auditory stimuli

During the presentation of visual stimuli the infants were sometimes presented with auditory stimuli (figure 1). These stimuli were presented at the onset of two of every three of the trials. The content and duration of the social videos (9–12 s) were not synchronized with the auditory stimuli. Each auditory stimulus presentation lasted 8 s and consisted of four different sounds (of vocal or non-vocal stimuli) presented for 0.37–2.92 s each, interleaved by short silence periods (of 0.16–0.24 s). The two auditory conditions were equivalent in terms of average sound intensity and duration (*p* > 0.65). Within the vocal condition, infants were presented with non-speech adult vocalizations of two speakers (who coughed, yawned, laughed and cried). Within the non-vocal condition, the infants were presented with naturalistic environmental sounds (that were not human- or animal-produced, but were likely to be familiar to infants of this age: running water, rattles, squeaky toys). Vocal and non-vocal stimuli were chosen from the Montreal Affective Voices (for more detail, see [47]) and the stimuli of the voice functional localizer (http://vnl.psy.gla.ac.uk/resources_main.php). Additional non-vocal stimuli (toy sounds) were also recorded by the authors [23].

### (c) Data processing and analysis

Changes in HbO_2_ and HHb chromophore concentration (μmol) were calculated and used as haemodynamic indicators of neural activity [48]. Initially, the recorded near-infrared attenuation measurements for each infant were analysed, and trials or channels were rejected from further analysis by looking time measures (trials were coded offline by a researcher unfamiliar with the study's aims: >60% trial looking considered valid) and the quality of the signals, using artefact detection algorithms [34,40]. For each infant, the trials and channels that survived these rejection criteria were entered into further analyses. Inclusion criteria required each channel to contain valid data in all three conditions. A minimum of three valid trials per condition was set as a threshold for inclusion within infants. Grand averaged time response curves of the haemodynamic responses (across all infants) for each channel were compiled. A time window was selected between 8 and 16 s post-stimulus onset for each trial. This period of time was selected to include the range of maximum concentration changes observed across infants for HbO_2_ and HHb, as illustrated by the example haemodynamic time courses provided in the electronic supplementary material. Either a significant increase in HbO_2_ concentration or a significant decrease in HHb is commonly accepted as an indicator of cortical activation in infant work [34]. During statistical analyses, if HbO_2_ and HHb were either to increase or decrease significantly in unison, the signal was considered inconsistent with a haemodynamic response to functional activation [48] and not reported in the analyses (for further discussion of physiological changes reported in infant fNIRS work see [25,34,35]).

## 3. Results

The high-risk and low-risk groups did not differ on a number of baseline measures, including age, gender, developmental stage, looking time measures and motion artefact detected in the fNIRS signal (table 1). The haemodynamic responses (μmol) within the two groups of infants were first assessed separately. For each channel, the maximum change (or amplitude) in HbO_2_ (increase in chromophore concentration) and/or HHb (decrease in chromophore concentration) was assessed during the specified time window (see §2). Group analyses were conducted on channels that showed a significant response to the social stimuli (either visual or auditory) in the channel-by-channel analyses. Given the exploratory nature of this study, *p*-values were uncorrected for multiple comparisons.

### (a) Visual social condition

**Figure 2.**
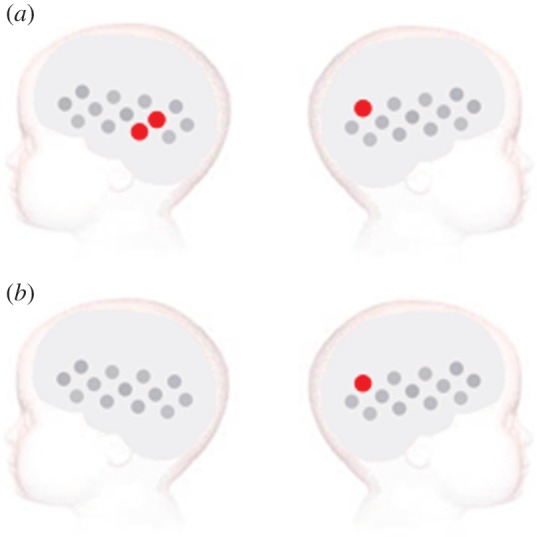
Visual social versus non-social stimuli analysis for the (*a*) low-risk and (*b*) high-risk infants. The statistically significant effects (two-tailed, *p* < 0.05) for the analysis of the visual social condition (no auditory stimulation) versus non-social baseline are presented on a diagram of the infant head. The channels that revealed a significant response during the specified time window of activation are plotted in red (increase in HbO_2_ concentration). Channels are plotted following the same layout as in figure 1.

To assess the responses to the visual social stimuli the experimental condition with no sound (visual only) was analysed relative to the baseline (*t*-test, two-tailed). This analysis revealed significant haemodynamic increases in HbO_2_ for both the high-risk and low-risk groups. These were centred over the posterior area of the arrays (figure 2; electronic supplementary material), corresponding to the posterior STS region of the cortex. The response in the low-risk group was more extensive, with several channels revealing a significant response (channels 8, 10 and 25; also a trend towards significance in channel 6: *t* = 2.17, *p* = 0.051; channel 12: *t* = 2.03, *p* = 0.061; and channel 13: *t* = 2.11, *p* = 0.057). By contrast, a response was only evident in one channel in the right array in the high-risk group (channel 25; also a trend towards significance in channel 26: *t* = 2.04, *p* = 0.059). This analysis did not reveal any significant decreases in HHb in either group.

In a confirmatory analysis, the average amplitude in the HbO_2_ concentration change in response to the visual social condition was compared across the two groups using a two-tailed independent-samples *t*-test in channels 8, 10 and 25. Channel 10 revealed a significantly greater response in the low-risk group compared with the high-risk group (*t* = 3.255, *p* = 0.003), and there was a trend towards significance in channel 8 (*t* = 1.934, *p* = 0.063). Grand averaged haemodynamic responses of channel 10 and 25 for each group are available in the electronic supplementary material. These two posterior temporal channels were chosen to provide an example of one channel that showed significant responses to the visual social stimuli in both groups (channel 25), and one channel that showed a greater response in the low-risk group compared with the high-risk group (channel 10).

### (b) Auditory vocal and non-vocal conditions

**Figure 3.**
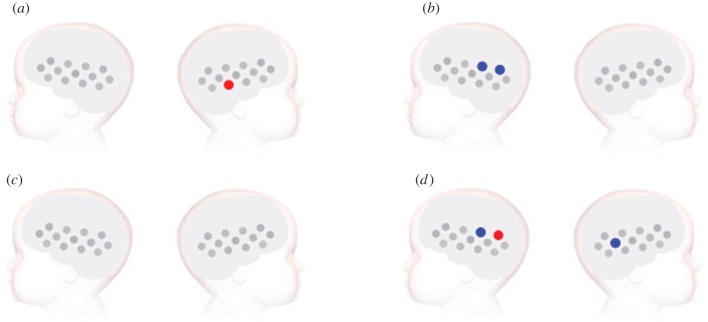
Vocal versus non-vocal stimuli analysis for the (*a*,*b*) low-risk and (*c*,*d*) high-risk infants. The statistically significant effects (two-tailed, *p* < 0.05) are displayed for (*a*,*c*) the vocal > non-vocal and (*b*,*d*) the non-vocal > vocal selective responses. The channels that revealed a significantly greater response during the specified time window of activation are plotted in red (increase in HbO_2_ concentration) and blue (decrease in HHb concentration). Channels are plotted following the same layout as in figure 1.

Paired-sample channel-by-channel *t*-tests (two-tailed) were performed within each group to compare responses to the vocal relative to the non-vocal condition (figure 3; electronic supplementary material). For statistical analyses of vocal and non-vocal auditory responses compared with silence (baseline), see the electronic supplementary material.

For the low-risk group this analysis revealed a greater hemodynamic response to the vocal condition relative to the non-vocal condition in the right hemisphere, centred over the anterior portion of the STS region (HbO_2_, channel 21: *t* = 2.42, *p* = 0.03). There was also a trend towards significance in channel 24 (HbO_2_, *t* = 1.80, *p* = 0.096) and in the left hemisphere in channel 5 (HHb, *t* = 1.90, *p* = 0.086). Further, the analysis revealed that the left posterior region of the array displayed greater haemodynamic responses to the non-vocal condition relative to the vocal condition (HHb, channel 9: *t* = 3.16, *p* = 0.007; channel 12: *t* = 2.35, *p* = 0.034). These responses were centred approximately over the mid-posterior STS region.

By contrast, for the high-risk group significant effects were only evident for the non-vocal condition relative to the vocal condition (HbO_2_, channel 12: *t* = 3.20, *p* = 0.006; HHb, channel 9: *t* = 2.91, *p* = 0.01, channel 23: *t* = 2.90, *p* = 0.01). This effect was largely confined to the posterior regions of the lateral arrays. There was also a trend towards significance in a further four channels (HbO_2_, channel 1: *t* = 1.91, *p* = 0.078; channel 15: *t* = 1.80, *p* = 0.094; channel 22: *t* = 1.95, *p* = 0.07; HHb, channel 19: *t* = 2.14, *p* = 0.05). In comparison with the low-risk group these responses were bilateral, and centred approximately over the mid-posterior STS region.

In a confirmatory analysis, vocal selectivity was compared across the two groups using a two-tailed independent-samples *t*-test (in the channel that was found to be vocal selective in the low-risk group—channel 21). In the right temporal region, this revealed a trend towards significance (HbO_2_, channel 21: *t* = 1.61, *p* = 0.12). Interestingly, in channel 24, which revealed a trend towards vocal selectivity in the low-risk group, a significantly greater HbO_2_ vocal-selective response was found in the low-risk group compared with the high-risk group (*t* = 2.48, *p* = 0.019).

## 4. Discussion

In this fNIRS study, infants at familial high risk for autism revealed different responses to social auditory and visual stimuli compared with a group of low-risk infants. The perception of the visual social stimuli produced a diminished response in the high-risk infants (one channel over the right posterior temporal cortex) relative to the low-risk infants (three channels over bilateral posterior temporal cortex), with significantly stronger responses evident in the low-risk infants relative to high-risk infants in the left STS region of the cortex. Further, in contrast to the low-risk infants, there was a noticeable absence of vocal-specialized areas in the high-risk group, with greater vocal selectivity in the right mid-posterior STS region in low-risk infants compared with high-risk infants. In contrast, the pattern of non-vocal selective responses was similar across groups. Indeed, non-vocal selective responses were more robust in the high-risk infants compared with the low-risk infants. In contrast to the high-risk group, the social (visual and auditory) responses in the low-risk infants were similar to those observed in previous research with infants [23,25,40,41,49] and adults [10,42,50]. These findings support our hypothesis that some infants at risk for autism may show a lack of cortical specialization to social stimuli within the first 6 months of life.

These group differences are not due to levels of overall looking time to the stimuli, head size, developmental stage or age, as these did not differ across the groups. Although there was a trend towards a higher number of channels rejected through the artefact detection algorithms in the high-risk group, this effect was driven largely by two infants, and all other measures of data quality were equal (table 1). As the differential response to the non-vocal sound was, if anything, stronger in the high-risk group, the sensitivity to stimulus-dependent effects on cortical activation appears comparable between the groups.

As stated in §1, it has been proposed that atypicality in one or more of the mechanisms that bias infants to orient towards and attend to socially relevant information from early in life may disrupt the typical developmental trajectory that leads to the adult social brain network [13–15]. We have found that areas of the mid-posterior STS region—which may form part of the developing social brain network—show a less specialized and/or weaker pattern of activation during the perception of complex and dynamic social stimuli relative to age-matched controls. Our findings are in line with fMRI research in children [6] and adults with autism [51,52], which report atypical functioning to the perception of biological motion and social stimuli in the posterior STS. Interestingly, the posterior STS activation to the perception of biological motion in the children with autism [6] was related to the degree of severity of autistic symptoms within individuals. It will therefore be of importance to revisit the current results when the high-risk infants in our study have been assessed for autism at 3 years of age, to allow us to ascertain whether STS region activation during infancy is associated with later outcome (autism or broader autism phenotype). Clearly, only a minority of our infants at risk will go on to a later diagnosis of autism and, given that recurrence rates vary according to infant (male/female) and family (simplex/multiplex status) [4], larger samples may be required in future studies to reliably detect predictive risk markers. In this regard, it may be important to note that Kaiser *et al.* [6] report that the unaffected siblings of children with autism share common patterns of atypical activation (‘trait activity’) in response to viewing biological motion in several cortical regions, including the right inferior temporal gyrus. Thus, it is possible that our current fNIRS results reflect ‘trait’ activity in our at-risk infants that will result in autism only when combined with other genetic, neural or environmental factors.

An important feature of the social stimuli we used may be that they were dynamic in the sense of rapidly changing over time. Taken together with our previous studies with different cohorts of at-risk infants with a different measure of brain function, namely EEG [16,21], a pattern of results is emerging that supports the view that the rapid temporal processing required by dynamic stimuli is affected in infants at risk and/or those who go on to a later diagnosis. A characteristic of interactions with other humans is that it is dynamic and probabilistic, and it may be these features that lead to greater deficits in social perception and cognition than in understanding of the physical world.

We acknowledge the possibility of cross-modal effects given our experimental design. Though we were careful to ensure the visual and auditory stimuli were non-synchronous and pseudo-randomized, our design was clearly restricted by what is possible with infants in a limited time period. However, we do not believe that cross-modal effects are a significant contributor to our findings as the voice-selective effects in the low-risk group largely replicate those of previous fMRI and fNIRS studies in adults and infants (see summary in [25]). In these previous studies the response is evident in the temporal cortex whether the auditory stimuli are [25,49] or are not [23,42] accompanied by visual stimuli. Further, the multi-modal presentation in Grossman *et al*. [24] used non-human dynamic visual stimuli alongside the vocal and non-vocal auditory stimuli, yet still found similar patterns of voice-selective activation to the current study.

In order to avoid a type II error in this initial study, we did not use correction for multiple comparisons. While Bonferroni correction is a rather conservative approach for such exploratory infant data, other methods such as Monte Carlo simulation, non-parametric statistics [53] or spatially contiguous activation [41]—the statistical likelihood of two or more spatially contiguous (neighbouring) channels producing false positive results is far lower—may be introduced in future work. However, in the present study we prefer to give a full account of the results, given that this is the first investigation of its kind. Indeed, if, for example, we were to remove single active channels, it would remove all social stimulus-specific activation in the high-risk group, presenting an even stronger case for atypical cortical activation in the infants at risk for autism.

A caveat of the current findings does exist. Given that the fNIRS headgear in the current study was restricted to the investigation of the frontal and temporal lobes, we cannot be certain whether the high-risk infants were responding less to the social stimuli, or whether they instead used an atypical network of brain regions not covered by the current set-up. Future work with a more extensive array of fNIRS channels or functional MRI (for the auditory contrasts) may help elucidate this further. Furthermore, there have been several MRI studies on anatomical brain development in individuals at risk for autism. Recent findings [39] suggest that white matter pathways may have aberrant development from 6–24 months in at-risk infants that go on to develop autism, in contrast to those who do not. Further, brain volume has been reported to be significantly enlarged by 2–3 years of age in children with autism relative to age-matched controls [54–59], and connectivity to be disrupted at 5 years of age [60,61]. Future anatomical work in younger infants will be essential to elucidate how the current functional data in at-risk infants may relate to atypical anatomical development, and whether one precedes or causes the other.

Taken together, our results are consistent with the view that atypical functioning of parts of the social brain network may be manifest from the first few months of life in infants at risk for a later diagnosis of autism. However, it is also possible that we have detected early manifestations of the broader autism phenotype, trait activity or adaptive responses in infants who will later go on to be unaffected [6]. It is likely that early atypical responses to social stimuli combine with other factors, consequently resulting in a later diagnosis of autism for some individuals [16].

## References

[1] Elsabbagh M Johnson MH 2010 Getting answers from babies about autism. Trends Cogn. Sci. 14, 81–87 10.1016/j.tics.2009.12.005 (doi:10.1016/j.tics.2009.12.005) 20074996

[2] Yirmiya N Charman T 2010 The prodrome of autism: early behavioral and biological signs, regression, peri- and post-natal development and genetics. Child Psychol. Psychiatry 51, 432–458 10.1111/j.1469-7610.2010.02214.x (doi:10.1111/j.1469-7610.2010.02214.x) 20085609

[3] Zwaigenbaum L 2007 Studying the emergence of autism spectrum disorders in high-risk infants: methodological and practical issues. J. Autism Dev. Disord. 37, 466–480 10.1007/s10803-006-0179-x (doi:10.1007/s10803-006-0179-x) 16897376

[4] Ozonoff S 2011 Recurrence risk for autism spectrum disorders: a baby siblings research consortium study. Pediatrics 128, e488–e495 21844053 10.1542/peds.2010-2825 PMC3164092

[5] Constantino JN Zhang Y Frazier T Abbacchi AM Law P 2010 Sibling recurrence and the genetic epidemiology of autism. Am. J. Psychiatry 167, 1349–1356 10.1176/appi.ajp.2010.09101470 (doi:10.1176/appi.ajp.2010.09101470) 20889652 PMC2970737

[6] Kaiser MD 2010 Neural signatures of autism. Proc. Natl Acad. Syst. 107, 21 223–21 228 10.1073/pnas.1010412107 (doi:10.1073/pnas.1010412107) PMC3000300 21078973

[7] Gottesman II Gould TD 2003 The endophenotype concept in psychiatry: etymology and strategic intentions. Am. J. Psychiatry 160, 636–645 10.1176/appi.ajp.160.4.636 (doi:10.1176/appi.ajp.160.4.636) 12668349

[8] Rommelse NNJ Geurts HM Franke B Buitelaar JK Hartman CA 2011 A review on cognitive and brain endophenotypes that may be common in autism spectrum disorder and attention-deficit/hyperactivity disorder and facilitate the search for pleiotropic genes. Neurosci. Biobehav. Rev. 35, 1363–1396 10.1016/j.neubiorev.2011.02.015 (doi:10.1016/j.neubiorev.2011.02.015) 21382410

[9] Rogers SJ 2009 What are infant siblings teaching us about autism in infancy? Autism Res. 2, 125–137 10.1002/aur.81 (doi:10.1002/aur.81) 19582867 PMC2791538

[10] Allison T Puce A McCarthy G 2000 Social perception from visual cues: role of the STS region. Trends Cogn. Sci. 4, 267–278 10.1016/S1364-6613(00)01501-1 (doi:10.1016/S1364-6613(00)01501-1) 10859571

[11] Pelphrey KA Carter EJ 2008 Charting the typical and atypical development of the social brain. Dev. Psychopathol. 20, 1081–1102 10.1017/S0954579408000515 (doi:10.1017/S0954579408000515) 18838032

[12] Adolphs R 2003 Cognitive neuroscience of human social behaviour. Nat. Rev. Neurosci. 4, 165–178 10.1038/nrn1056 (doi:10.1038/nrn1056) 12612630

[13] Dawson G Webb SJ McPartland J 2005 Understanding the nature of face processing impairment in autism: insights from behavioral and electrophysiological studies. Dev. Neuropsychol. 27, 403–424 10.1207/s15326942dn2703_6 (doi:10.1207/s15326942dn2703_6) 15843104

[14] Johnson MH Griffin R Csibra G Halit H Farroni T de Haan M Tucker LA Baron-Cohen S Richards J 2005 The emergence of the social brain network: evidence from typical and atypical development. Dev. Psychopathol. 17, 599–619 10.1017/S0954579405050297 (doi:10.1017/S0954579405050297) 16262984 PMC1464100

[15] Schultz RT 2005 Developmental deficits in social perception in autism: the role of the amygdala and fusiform face area. Int. J. Dev. Neurosci. 23, 125–141 10.1016/j.ijdevneu.2004.12.012 (doi:10.1016/j.ijdevneu.2004.12.012) 15749240

[16] Elsabbagh M 2012 Infant neural sensitivity to dynamic eye gaze is associated with later emerging autism. Curr. Biol. 22, 338–342 10.1016/j.cub.2011.12.056 (doi:10.1016/j.cub.2011.12.056) 22285033 PMC3314921

[17] Johnson MH 2001 Functional brain development in humans. Nat. Rev. Neurosci. 2, 475–483 10.1038/35081509 (doi:10.1038/35081509) 11433372

[18] Johnson MH 2011 Interactive specialization: a domain-general framework for human functional brain development? Dev. Cogn. Neurosci. 1, 7–21 10.1016/j.dcn.2010.07.003 (doi:10.1016/j.dcn.2010.07.003) 22436416 PMC6987575

[19] Elsabbagh M 2009 Neural correlates of eye gaze processing in the infant broader autism phenotype. Biol. Psychiatry 65, 31–38 10.1016/j.biopsych.2008.09.034 (doi:10.1016/j.biopsych.2008.09.034) 19064038

[20] McCleery JP Akshoomoff N Dobkins KR Carver LJ 2009 Atypical face versus object processing and hemispheric asymmetries in 10-month-old infants at risk for autism. Biol. Psychiatry 66, 950–957 10.1016/j.biopsych.2009.07.031 (doi:10.1016/j.biopsych.2009.07.031) 19765688 PMC2783702

[21] Guiraud JA Kushnerenko E Tomalski P Davies K Ribeiro H Johnson MH, BASIS Team 2011 Differential habituation to repeated sounds in infants at high risk for autism. Neuroreport 22, 845 21934535 10.1097/WNR.0b013e32834c0bec

[22] Luyster RJ Wagner JB Vogel-Farley V Tager-Flusberg H Nelson CA 2011 Neural correlates of familiar and unfamiliar face processing in infants at risk for autism spectrum disorders. Brain Topogr. 24, 220–228 10.1007/s10548-011-0176-z (doi:10.1007/s10548-011-0176-z) 21442325 PMC3171602

[23] Blasi A 2011 Early specialization for voice and emotion processing in the infant brain. Curr. Biol. 21, 1220–1224 10.1016/j.cub.2011.06.009 (doi:10.1016/j.cub.2011.06.009) 21723130

[24] Grossmann T Oberecker R Koch SP Friederici AD 2010 The developmental origins of voice processing in the human brain. Neuron 65, 852–858 10.1016/j.neuron.2010.03.001 (doi:10.1016/j.neuron.2010.03.001) 20346760 PMC2852650

[25] Lloyd-Fox S Blasi A Mercure E Elwell CE Johnson MH 2011 The emergence of cerebral specialization for the human voice over the first months of life. Soc. Neurosci. 7, 317–330 10.1080/17470919.2011.614696 (doi:10.1080/17470919.2011.614696) 21950945

[26] Minagawa-Kawai Y 2011 Optical brain imaging reveals general auditory and language-specific processing in early infant development. Cereb. Cortex 21, 254–261 10.1093/cercor/bhq082 (doi:10.1093/cercor/bhq082) 20497946 PMC3020578

[27] Gervais H 2004 Abnormal cortical voice processing in autism. Nat. Neurosci. 7, 801–802 10.1038/nn1291 (doi:10.1038/nn1291) 15258587

[28] Rutherford M Baron-Cohen S Wheelwright S 2002 Reading the mind in the voice: a study with normal adults and adults with Asperger syndrome and high functioning autism. J. Autism Dev. Disord. 32, 189–194 10.1023/A:1015497629971 (doi:10.1023/A:1015497629971) 12108620

[29] Klin A 1991 Young autistic children's listening preferences in regard to speech: a possible characterization of the symptom of social withdrawal. J. Autism Dev. Disord. 21, 29–42 10.1007/BF02206995 (doi:10.1007/BF02206995) 1828067

[30] Čeponienė R Lepistö T Shestakova A Vanhala R Alku P Näätänen R Yaguchi K 2003 Speech–sound-selective auditory impairment in children with autism: they can perceive but do not attend. Proc. Natl Acad. Sci. USA 100, 5567 10.1073/pnas.0835631100 (doi:10.1073/pnas.0835631100) 12702776 PMC154385

[31] Eyler LT Pierce K Courchesne E 2012 A failure of left temporal cortex to specialize for language is an early emerging and fundamental property of autism. Brain 135, 949–960 10.1093/brain/awr364 (doi:10.1093/brain/awr364) 22350062 PMC3286331

[32] Whittingstall K Stroink G Gates L Connolly JF Finley A 2003 Effects of dipole position, orientation and noise on the accuracy of EEG source localization. BioMed. Eng. OnLine 2, 14 10.1186/1475-925X-2-14 (doi:10.1186/1475-925X-2-14) 12807534 PMC166138

[33] Richards JE 2005 Localizing cortical sources of event-related potentials in infants’ covert orienting. Dev. Sci. 8, 255–278 10.1111/j.1467-7687.2005.00414.x (doi:10.1111/j.1467-7687.2005.00414.x) 15819757 PMC1464102

[34] Lloyd-Fox S Blasi A Elwell CE 2010 Illuminating the developing brain: the past, present and future of functional near infrared spectroscopy. Neurosci. Biobehav. Rev. 34, 269–284 10.1016/j.neubiorev.2009.07.008 (doi:10.1016/j.neubiorev.2009.07.008) 19632270

[35] Gervain J Mehler J Werker JF Nelson CA Csibra G Lloyd-Fox S Shukla M Aslin RN 2011 Near-infrared spectroscopy: a report from the McDonnell infant methodology consortium. Dev. Cogn. Neurosci. 1, 22–46 10.1016/j.dcn.2010.07.004 (doi:10.1016/j.dcn.2010.07.004) 22436417 PMC6987576

[36] Aslin RN Mehler J 2005 Near-infrared spectroscopy for functional studies of brain activity in human infants: promise, prospects, and challenges. J. Biomed. Opt. 10, 011009 10.1117/1.1854672 (doi:10.1117/1.1854672) 15847575

[37] Fukui Y Ajichi Y Okada E 2003 Monte Carlo prediction of near-infrared light propagation in realistic adult and neonatal head models. Appl. Opt. 42, 2881–2887 10.1364/AO.42.002881 (doi:10.1364/AO.42.002881) 12790436

[38] Steinbrink J Villringer A Kempf F Haux D Boden S Obrig H 2006 Illuminating the BOLD signal: combined fMRI-fNIRS studies. Magn. Reson. Imaging 24, 495–505 10.1016/j.mri.2005.12.034 (doi:10.1016/j.mri.2005.12.034) 16677956

[39] Wolff JJ 2012 Differences in white matter fiber tract development present from 6 to 24 months in infants with autism. Am. J. Psychiatry. 169, 589–600 10.1176/appi.ajp.2011.11091447 (doi:10.1176/appi.ajp.2011.11091447) 22362397 PMC3377782

[40] Lloyd-Fox S Blasi A Volein A Everdell N Elwell CE Johnson MH 2009 Social perception in infancy: a near infrared spectroscopy study. Child Dev. 80, 986–999 10.1111/j.1467-8624.2009.01312.x (doi:10.1111/j.1467-8624.2009.01312.x) 19630889

[41] Lloyd-Fox S Blasi A Everdell N Elwell CE Johnson MH 2011 Selective cortical mapping of biological motion processing in young infants. J. Cogn. Neurosci. 23, 2521–2532 10.1162/jocn.2010.21598 (doi:10.1162/jocn.2010.21598) 20954934

[42] Belin P Zatorre RJ Lafaille P Ahad P Pike B 2000 Voice-selective areas in human auditory cortex. Nature 403, 309–312 10.1038/35002078 (doi:10.1038/35002078) 10659849

[43] World Health Organization 1993 Mental disorders: glossary and guide to their classification in accordance with the 10th revision of the international classification of diseases: research diagnostic criteria. Geneva, Switzerland: World Health Organization

[44] Mullen EM 1995 Mullen scales of early learning. Circle Pines, MN: American Guidance Service

[45] Everdell NL Gibson AP Tullis IDC Vaithianathan T Hebden JC Delpy DT 2005 A frequency multiplexed near-infrared topography system for imaging functional activation in the brain. Rev. Sci. Instrum. 76, 093705 10.1063/1.2038567 (doi:10.1063/1.2038567)

[46] Salamon G Raynaud C Regis J Rumeau C 1990 Magnetic resonance imaging of the pediatric brain. New York, NY: Raven Press

[47] Belin P Fillion-Bilodeau S Gosselin F 2008 The montreal affective voices: a validated set of nonverbal affect bursts for research on auditory affective processing. Behav. Res. Method 40, 531 10.3758/BRM.40.2.531 (doi:10.3758/BRM.40.2.531) 18522064

[48] Obrig H Villringer A 2003 Beyond the visible—imaging the human brain with light. J. Cereb. Blood Flow Metab. 23, 1–18 10.1097/00004647-200301000-00001 (doi:10.1097/00004647-200301000-00001) 12500086

[49] Grossmann T Johnson MH Lloyd-Fox S Blasi A Deligianni F Elwell C Csibra G 2008 Early cortical specialization for face-to-face communication in human infants. Proc. R. Soc. B 275, 2803–2811 10.1098/rspb.2008.0986 (doi:10.1098/rspb.2008.0986) PMC2572680 18755668

[50] Pelphrey K Morris J Michelich C Allison T McCarthy G 2005 Functional anatomy of biological motion perception in posterior temporal cortex: an fMRI study of eye, mouth and hand movements. Cereb. Cortex 15, 1866–1876 10.1093/cercor/bhi064 (doi:10.1093/cercor/bhi064) 15746001

[51] Pelphrey KA Morris JP McCarthy G 2005 Neural basis of eye gaze processing deficits in autism. Brain 128, 1038 10.1093/brain/awh404 (doi:10.1093/brain/awh404) 15758039

[52] Pelphrey KA Shultz S Hudac CM Vander Wyk BC 2011 Research review: constraining heterogeneity: the social brain and its development in autism spectrum disorder. J. Child Psychol. Psychiatry 52, 631–644 10.1111/j.1469-7610.2010.02349.x (doi:10.1111/j.1469-7610.2010.02349.x) 21244421 PMC3096715

[53] Maris E Oostenveld R 2007 Nonparametric statistical testing of EEG- and MEG-data. J. Neurosci. Methods 164, 177–190 10.1016/j.jneumeth.2007.03.024 (doi:10.1016/j.jneumeth.2007.03.024) 17517438

[54] Courchesne E 2001 Unusual brain growth patterns in early life in patients with autistic disorder. Neurology 57, 245–254 10.1212/WNL.57.2.245 (doi:10.1212/WNL.57.2.245) 11468308

[55] Courchesne E Mouton PR Calhoun ME Semendeferi K Ahrens-Barbeau C Hallet MJ Barnes CC Pierce K 2011 Neuron number and size in prefrontal cortex of children with autism. JAMA 306, 2001–2010 10.1001/jama.2011.1638 (doi:10.1001/jama.2011.1638) 22068992

[56] Hazlett HC 2005 Magnetic resonance imaging and head circumference study of brain size in autism: birth through age 2 years. Arch. Gen. Psychiatry 62, 1366 10.1001/archpsyc.62.12.1366 (doi:10.1001/archpsyc.62.12.1366) 16330725

[57] Hazlett HC Poe MD Gerig G Styner M Chappell C Smith RG Vachet C Piven J 2011 Early brain overgrowth in autism associated with an increase in cortical surface area before age 2 years. Arch. Gen. Psychiatry 68, 467 10.1001/archgenpsychiatry.2011.39 (doi:10.1001/archgenpsychiatry.2011.39) 21536976 PMC3315057

[58] Sparks B 2002 Brain structural abnormalities in young children with autism spectrum disorder. Neurology 59, 184–192 10.1212/WNL.59.2.184 (doi:10.1212/WNL.59.2.184) 12136055

[59] Schumann CM 2010 Longitudinal magnetic resonance imaging study of cortical development through early childhood in autism. J. Neurosci. 30, 4419 10.1523/JNEUROSCI.5714-09.2010 (doi:10.1523/JNEUROSCI.5714-09.2010) 20335478 PMC2859218

[60] Dinstein I 2011 Disrupted neural synchronization in toddlers with autism. Neuron 70, 1218–1225 10.1016/j.neuron.2011.04.018 (doi:10.1016/j.neuron.2011.04.018) 21689606 PMC3119852

[61] Di Martino A Kelly C Grzadzinski R Zuo XN Mennes M Mairena MA Lord C Castellanos FX Milham MP 2011 Aberrant striatal functional connectivity in children with autism. Biol. Psychiatry 69, 847–856 10.1016/j.biopsych.2010.10.029 (doi:10.1016/j.biopsych.2010.10.029) 21195388 PMC3091619

